# Severe malnutrition with and without HIV-1 infection in hospitalised children in Kampala, Uganda: differences in clinical features, haematological findings and CD4^+ ^cell counts

**DOI:** 10.1186/1475-2891-5-27

**Published:** 2006-10-16

**Authors:** Hanifa Bachou, Thorkild Tylleskär, Robert Downing, James K Tumwine

**Affiliations:** 1 Department of Paediatrics and Child Health, Makerere University Medical School, P O Box 7072, Kampala, Uganda; 2 Centre for International Health, University of Bergen, Norway; 3 CDC/Uganda Virus Research Institute Research Collaboration, P O Box 49, Entebbe, Uganda

## Abstract

**Background:**

The aim of this study was to describe the clinical features, haematological findings and CD4^+ ^and CD8^+ ^cell counts of severely malnourished children in relation to human immunodeficiency virus (HIV) infection.

**Methods:**

The study was conducted in the paediatric wards of Mulago hospital, which is Uganda's national referral and teaching hospital. We studied 315 severely malnourished children (presence of oedema and/or weight-for-height: z-score < -3) and have presented our findings. At admission, the CD4^+ ^and CD8^+ ^cells were measured by the flow cytometry and HIV serology was confirmed by Enzyme linked Immunoassay for children >18 months of age, and RNA PCR was performed for those ≤18 months. Complete blood count, including differential counts, was determined using a Beckman Coulter counter.

**Results:**

Among the 315 children, 119 (38%) were female; the median age of these children was 17 months (Interquartile range 12–24 months), and no difference was observed in the HIV status with regard to gender or age. The children showed a high prevalence of infections: pneumonia (68%), diarrhoea (38%), urinary tract infection (26%) and bacteraemia (18%), with no significant difference with regard to the HIV status (HIV-positive versus HIV-negative children). However, the HIV-positive children were more likely to have persistent diarrhoea than the HIV-uninfected severely malnourished children (odds ratio (OR) 2.0, 95% confidence interval (CI) 1.2–3.6). When compared with the HIV-negative children, the HIV-positive children showed a significantly lower median white blood cell count (10700 versus 8700) and lymphocyte count (4033 versus 2687). The CD4^+ ^cell percentages were more likely to be lower in children with non-oedematous malnutrition than in those with oedematous malnutrition even after controlling for the HIV infection.

The novel observation of this study is that the CD4^+ ^percentages in both HIV-positive and HIV-negative children without oedema were lower that those in children with oedema. These observations appear to imply that the development of oedema requires a certain degree of immunocompetence, which is an interesting clue to the pathophysiology of oedema in severe malnutrition.

## Background

Severe malnutrition has been associated with acquired immunodeficiency (AID) among children worldwide, and it is referred to as Nutritionally Acquired Immunodeficiency Syndrome or NAIDS [1,2]. With the advent of the human immunodeficiency virus (HIV) pandemic, there has been a tendency to overlook the role of malnutrition in immunodeficiency, and indeed, only a handful of studies have investigated the CD4^+ ^and CD8^+ ^lymphocyte subsets in severely malnourished children [3,4].

There is little information on the effect of the added burden of HIV infection on the clinical features [5-7] and cellular immunity of severely malnourished children. The objective of this study was to report the clinical features, haematological findings and CD4^+ ^and CD8^+ ^lymphocyte subsets of severely malnourished children with regard to their HIV status.

## Subjects and methods

**Figure 1:**
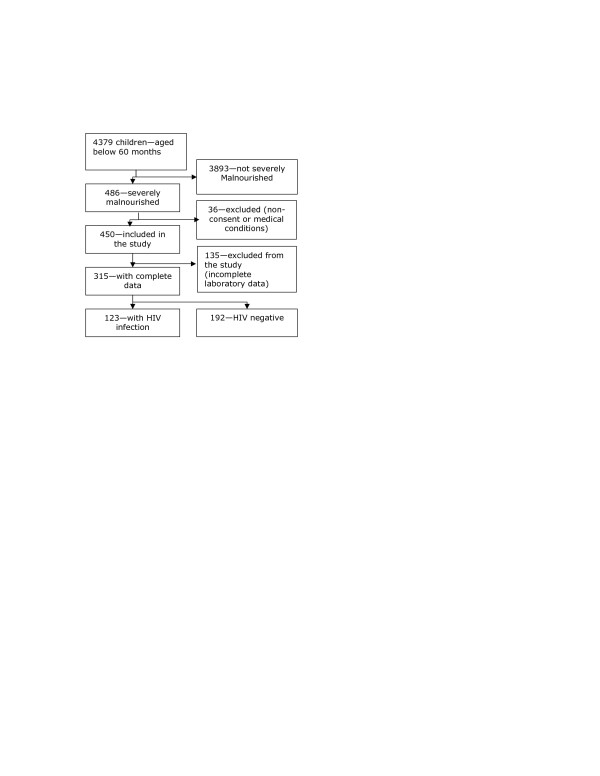
Study profile showing the enrolment process of the 315 children in the study.

All severely malnourished children consecutively admitted to the paediatric wards of Mulago hospital, which is Uganda's national referral and teaching hospital, during the two peak seasons of malnutrition, namely, September-November 2003 and September-December 2004 were followed up from the time of admission to outcome (death or discharge). In this study, we included a total of 450 severely malnourished children (presence of oedema and/or weight-for-height: z-score < -3) after obtaining the informed consent of their parents or caregivers (Figure 1); the age of these children was below 60 months. The risk factors for death in the first peak (2003) have been stated in a previous report that describes the methodology in greater detail.

In this paper, we report the complete results of the HIV tests as well as the CD4^+ ^and CD8^+ ^cell counts and percentages of the 315 children. In the case of 135 children, complete laboratory data could not be obtained; this was due to the lack of reagents in the case of 89 children; inadequate blood volume in 38, haemolysis in 6 and absence of blood sample in 2. The basic characteristics of the 315 children with complete results were compared with those of the 135 children with incomplete results.

The following parameters were recorded for all the children: demographic characteristics (age and sex), clinical features (weight, height/length and presence of oedema and diarrhoea), haematological tests (haemoglobin concentration, white blood cell (WBC) count and differentials and presence of malarial parasites), HIV tests (ELISA and RNA PCR), microbial tests (blood and urine culture and sensitivity), immunologic tests (CD4^+ ^and CD8^+ ^cell counts and percentages) and chest x-ray reports. We used the CD4^+ ^cell percentage to categorize children with or without the HIV infection. The clinical definition of malnourishment classified all our patients into category C because all the children were severely malnourished. The haemoglobin concentrations were evaluated according to the WHO criteria: <5 g/dL and <4 mg/dL are referred to as severe anaemia and very severe anaemia, respectively.

### Laboratory methods

Blood was collected in 5-ml EDTA vacutainer tubes (Becton Dickinson, Franklin Lakes, NJ, USA) in the mornings between 8–11 am by venipuncture and transported within 4 h to Uganda Virus Research Institute (UVRI) laboratory, Entebbe for serological testing. HIV testing was performed using the standard HIV algorithm of two enzyme-linked immunoassays (EIA) in parallel. Western blot and real-time polymerase chain reaction (RT-PCR) were performed to confirm a positive EIA test for children below 18 months of age and those with indeterminate results on EIA.

TriTEST reagents (CD3, FITC/CD4, PE/CD45, PerCP and CD3, FITC/CD8, PE/CD45, Per CP) were used to stain PBMC for CD4^+^/CD8^+ ^cell counting according to the manufacturer's instructions. FACScan instrument and MultiSET software were used to perform flow cytometry and report the absolute CD4^+ ^and CD8^+ ^cell counts of each sample by using the dual-platform approach (Becton Dickinson, Franklin Lakes, NJ, USA). Complete blood count, including differential counts, was assessed using a Beckman Coulter counter [8]. Blood was stained within 12 h of collection, and the observations were analysed within 24 h.

Severe malnutrition was defined according to the WHO classification and the presence of severe wasting (weight-for-height < 3 SD of the NCHS/WHO reference values with no oedema) and/or oedematous malnutrition (presence of symmetrical oedema involving at least the feet) [9]. The children were divided in two groups; HIV-positive and HIV-negative groups.

The study protocol was approved by the Regional Committee for Medical Ethics, Bergen, Norway (REK Vest), Makerere University Faculty of Medicine Ethics and Research Committee, Mulago Hospital Ethics Committee and the Uganda National Council for Science and Technology.

Statistical analysis was performed using SPSS version 13. Medians were used to calculate the central tendency and interquartile range (IQR) for the spread of haemoglobin concentration, WBC, total lymphocyte and CD4^+ ^and CD8^+ ^cell counts. Children were grouped by their gender (male or female), age in months (≤24 months and >24 months), presence or absence of oedematous malnutrition and HIV infection and CD4^+ ^levels (CD4^+ ^cell percentage < 20% and < 15%). Chi square and Wilcoxon-Mann-Whitney tests and multivariate analysis were used to determine differences with regard to the HIV status, gender and type of severe malnutrition (oedematous versus non-oedematous). A 2-tailed p value of < 0.05 was considered significant. Binary logistic regression models were constructed using the HIV status as the outcome variable. The appropriate important baseline data of clinical significance was included in a regression model and used for adjustment. The chi-square test was used to select variables according to their statistical significance (p < 0.05). Dummy variables were created for the categorical variables used. The chosen dependent variables were tested for interactions, and the very significant variables were stratified to assess for the possibility of effect modification. Positive interactions remained in the final model. Independent variables that showed a persistently non-significant relationship with the dependant variable during modelling were excluded from the final model.

## Results

Of the 315 children, 119 (38%) were female, and the median age of these children was 17.0 months (IQR 12–24). The age of half the children was between 12–24 months, and that of a few children (3%) was below 6 months (Table 1). The age distribution was not affected by their HIV status. Almost half the children (170/315) had oedematous malnutrition (kwashiorkor and marasmic-kwashiorkor). These characteristics (sex, age and type of malnutrition) were comparable to those of the 135 children with incomplete laboratory data.

**Table 1:** Characteristics of children aged below 60 months with severe malnutrition during 2 peak malnutrition periods.

Age group	HIV-positive children n = 123		HIV-uninfected children n = 192		Total
			
Months	Male	Female	Male	Female	
0–5.9	1	2	4	4	11
6–11.9	15	8	21	14	58
12–23.9	36	21	66	37	160
24–35.9	17	8	19	6	50
36–47.9	4	4	6	6	20
48–59.9	2	5	5	4	16
Total	75	48	121	71	315

HIV infection was detected in 123/315 children (approximately 40%). The HIV-infected children were less likely to present with oedema (OR 0.5, 95% CI 0.3–0.7). Of all the severely malnourished children, only 27 (9%) had no identifiable infection on admission, 51 (16%) had only one type of infection and the majority, that is, 227 (72%) had more than one type of infection on admission. The infections included pneumonia (68%), diarrhoea (38%), urinary tract infection (26%), bacteraemia (18%), malaria (9%) and oral thrush (11%) (Table 2). Overall, there was no significant difference in the prevalence of infection with regard to the HIV status. However, the HIV-1-infected children were more likely to have persistent diarrhoea and oral thrush (Table 2).

**Table 2:** Characteristics, co-existing medical conditions and diagnosis of children aged <60 months with severe malnutrition.

	HIV-positive children n (%)	HIV-negative children n (%)	Odds ratio (95% CI)
**Symptoms and signs**	**(n = 123)**	**(n = 192)**	
Diarrhoea (all)	52 (42)	67 (35)	1.4 (0.9–2.2)
Persistent diarrhoea (>2 weeks)	32 (26)	28 (15)	2.1 (1.1–3.8)*
Oral thrush	20 (16)	15 (8)	2.3 (1.1–4.7)*
Bilateral oedema (nutritional)	53 (43)	119 (62)	0.5 (0.3–0.7)*
Severe dehydration	7 (6)	11 (6)	1.0 (0.4–2.7)
**Chest x-ray findings**	**(n = 109)**	**(n = 158)**	
Bronchopneumonia	26 (24)	48 (30)	0.7 (0.4–1.3)
Interstitial pneumonia	40 (37)	48 (30)	1.3 (0.8–2.2)
Suspected tuberculosis	14 (13)	18 (11)	1.2 (0.5–2.4)
**Blood tests**	**(n = 122)**	**(n = 191)**	
Malarial parasites	10 (9)	19 (11)	0.9 (0.4–1.9)
Severe anaemia (Hb < 5 g/dL)	10 (8)	2 (6)	1.3 (0.6–3.3)
Bacteraemia	24 (20)	32 (17)	1.2 (0.7–2.2)
**Urine tests**	**(n = 109)**	**(n = 160)**	
Bacteruria	33 (30)	36 (23)	1.5 (0.9–2.6)

*Statistically significant

The median haemoglobin concentration of these children was below 9 g/dL. There was no significant difference in the haemoglobin concentration with regard to the type of severe malnutrition or HIV status (Table 3). The total WBC count was significantly lower in the HIV-positive children (8.9 × 10^6^; IQR 5.4–11.3) than in the HIV-negative children (9.1 × 10^6^; 7.2-3.5) (p = 0.028). Among the HIV-infected children, the total WBC count was lower in the non-oedematous children than in the oedematous children. However, this difference was not observed among the HIV-uninfected children (Table 4).

**Table 3:** Laboratory data of all severely malnourished children aged <60 months grouped by their HIV status.

	HIV-positive median (IQR) n = 123	HIV-negative median (IQR) n = 192
Haemoglobin	7.8 (6.4–9.2)	8.1 (6.5–9.6)
Total WBC (10^9^/L)	8.9 (5.4–11.3)	9.1 (7.2–13.5)**
Neutrophils (10^9^/L)	4.9 (2.8–8.0)	5.9 (3.4–8.9)
Neutrophils (%)	59 (35–71)	55 (41–65)
Monocytes (10^9^/L)	0.22 (0.11–0.94)	0.28 (0.15–0.53)
Monocytes (%)	2 (1.8–7.5)	2 (1.8–5)
Total lymphocytes (10^9^/L)	2.9 (2.1–4.9)	4.5 (2.9–6.3)**
Lymphocytes (%)	39 (26–50)	40 (32–50)
CD4^+ ^cell count (10^6^/L)	497 (280–1379)	1265 (829–1758)***
CD4^+ ^cells (%)	18 (12–34)	33 (26–40)***
CD8^+ ^cell count (10^6^/L)	880 (490–1750)	588 (331–913)***
CD8^+ ^cells (%)	31 (23–50)	15 (13–21)***
CD4^+^/CD8^+ ^ratio	0.76 (0.24–1.19)	2.0 (1.5–2.8)***

*p < 0.05, ** p < 0.01, and *** p < 0.001

**Table 4:** Laboratory data of <60-month-old severely malnourished children categorised by their HIV status and malnutrition type.

	HIV-positive children		HIV-negative children	
	Oedema n = 53 median (IQR)	No oedema n = 70 median (IQR)	Oedema n = 119 median (IQR)	No oedema n = 73 median (IQR)
Haemoglobin (g/dL)	8.2 (6.4–9.6)	7.3 (6–9.1)	8.0 (6.1–9.3)	8.4 (6.7–9.8)
White blood cells (10^9^/L)	11.0 (8.3–17)	7.2 (4.2–12)	10.0 (7.7–17)	11.0 (8.8–15)
Neutrophils (10^9^/L)	6.2 (3.1–8.5)*	2.9 (2.3–7.7)	5.4 (3.5–8.8)	6.1 (3.2–9.0)
Neutrophils (%)	59.0 (34–70)	61.0 (37–73)	55.0 (48–66)	53.0 (38–63)
Monocytes (10^9^/L)	667.0 (182–1246)*	153.0 (83–263)	217.0 (107–540)	412.0 (176–534)
Monocytes (%)	5.7 (2–10)*	2.0 (1.0–2.8)	2.0 (1–5)	3.5 (2–6.5)
Total lymphocytes (10^9^/L)	3.3 (2.4–6.3)	2.5 (1.7–4.1)*	4.5 (2.6–7.2)	4.4 (3.6–5.7)
Lymphocytes (%)	39.0 (23–57)	36.0 (26–50)	39.0 (31–49)	42.0 (32–55)
CD4^+ ^cell counts	630.0 (305–1759)***	379.0 (123–713)	1354 (894–1914)***	1169 (682–1600)
CD4^+ ^cells (%)	20.0 (14–42)***	14.0 (5–25)	35.0 (29–44)***	27.0 (22–37)
CD8^+ ^cell counts	1046 (521–1896)	811.0 (462–1363)	822.0 (492–1367)**	595 (328–1054)
CD8^+ ^cells (%)	23.0 (20–39)*	41.0 (27–56)	15.0 (12–21)	16.0 (13–21)
CD4^+^/CD8^+ ^cell ratio	0.9 (0.4–1.6)***	0.4 (0.1–0.9)	2.2 (1.6–3.0)	1.9 (1.2–2.8)

*p value < 0.05, **p value < 0.005, and ***p value < 0.001, comparing each HIV status group with the type of severe malnutrition.

The total lymphocyte counts were 2.9 × 10^9 ^(IQR 2.0–4.9) in the HIV-positive children and 4.5 × 10^9 ^(IQR 2.9–6.3) in the HIV-uninfected children (p = 0.008). The absolute lymphocyte counts were 2.7 × 10^9 ^(IQR 1.8–4.9) in the HIV-infected children and 4.0 × 10^9 ^(2.8–5.6) in the HIV-uninfected children (p < 0.001). The total lymphocyte, monocyte and neutrophil counts were lower in the HIV-positive children with no oedema than in those with oedema; this was not observed among the HIV-negative children.

**Figure 2:**
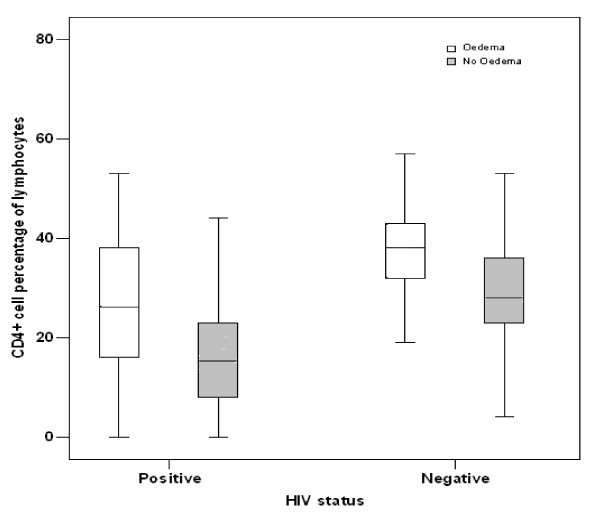
Box and whisker plot showing the median and the interquartile range of the percentages of CD4^+ ^cells in severely malnourished children who were grouped based on their HIV status and type of malnutrition.

Regardless of their HIV status, children with severe non-oedematous malnutrition (marasmus) had significantly lower CD4^+ ^count, CD4^+^and CD8^+ ^percentages and CD4^+^/CD8^+ ^ratios than those with oedematous malnutrition (kwashiorkor and marasmic-kwashiorkor) (Table 4 and Figure 2)

The CD4^+ ^cell percentage was below 25% in one-third of the 315 severely malnourished children. Among these one-third children, the CD4^+ ^cell percentage was between 15%–24% in 17% (55/315) children, indicating moderate cellular immunosuppression, while it was below 15% in 18% children, indicating severe cellular immunosuppression. Both these categories of cellular immunosuppression were present in HIV-infected and HIV-uninfected groups (Table 5). The CD4^+ ^cell percentages were more likely to be below 15% (OR 4.2, CI 1.4–12.6) and between 15%–25% (OR 2.0, CI 0.6 – 6.8) in children with non-oedematous malnutrition than in those with oedema. This difference persisted even after controlling for their HIV status (Figure 2).

**Table 5:** Distribution of all severely malnourished children by malnutrition type, cellular immunological category and HIV status.

	HIV-positive children n (%)	HIV-uninfected children n (%)	Total
**Oedema**	**n = 53**	**n = 119**	**n = 172**
CD4^+ ^≥ 25%*	28 (53)	119 (100)	171 (99)
CD4^+ ^15%–24%**	15 (28)	0 (0)	0 (0)
CD4^+ ^< 15%***	10 (19)	0 (0)	1 (1)
**No oedema**	**n = 70**	**n = 73**	**n = 143**
CD4^+ ^≥ 25%*	15 (21)	50 (69)	65 (45)
CD4^+ ^15%–24%**	22 (31)	15 (20)	37 (26)
CD4^+ ^< 15%***	33 (62)	8 (11)	41 (29)

*No evidence of suppression, **Evidence of moderate suppression, ***Severe suppression

## Discussion

In this study, we have reviewed an old problem – severe malnutrition in children – by using modern techniques for assessing the immunocompetence that has developed over the last decades in response to the HIV/AIDS pandemic. We have used the up-to-date laboratory techniques for the assessment of lymphocyte subsets in recognised laboratories. Since some of the severely malnourished children are also HIV positive, it is now possible to describe the clinical and laboratory features of the two groups of patients: severely malnourished children with HIV infection and those without HIV infection. We have noticed the well-known fact that the clinical features of severe malnutrition and HIV/AIDS overlap in young children. This affects the possibility of an accurate clinical diagnosis of HIV infection in settings with poor resources and often with inadequate HIV-testing facilities, particularly in cases wherein the two conditions, namely, malnutrition and HIV infection co-exist [16]. Therefore, a question that arises is whether there are any clinical differences that may raise a suspicion of HIV infection in cases of severe malnutrition?

In our study in Uganda, both the groups showed a high prevalence of multiple infections, including pneumonia, diarrhoea, bacteraemia, malaria, urinary tract infection and oral thrush. For respiratory, blood stream or urinary tract infections, no significant difference was observed with regard to the HIV status. The only two conditions that were over-represented among the HIV-positive children were persistent diarrhoea and oral thrush. In view of the remarkable difficulties encountered while clinically differentiating between malnutrition and HIV infection, we strongly support the establishment of routine counselling and testing for HIV-1 infection among paediatric patients with severe malnutrition in settings where HIV infection is an existing problem. In our study, we observed that there was a high acceptability of counselling and testing for HIV-1 infection; another reason for the absence of hesitation in organising routine counselling and testing for HIV-1 infection is that the study subjects were in the paediatric age group.

The drop-out cases in this study were mostly due to random factors, and we believe that these did not affect the selection of the study subjects in any systematic manner. In addition, the basic characteristics of the 315 of the 450 severely malnourished children analysed in this study were the same as those of the drop-out cases.

The median CD4^+ ^cell counts and percentages observed in this study were compared to the recently reported median CD4^+ ^counts and percentages of healthy Ugandan children younger than 5 years [18]. Among the HIV-uninfected children without oedema, as many as one-third had signs of immunosuppression with a CD4^+ ^percentage below 25%; this number was 1 in 12 in the HIV-uninfected children with oedema. Almost 80% of the HIV-positive children without oedema had signs of immunosuppression that was revealed as a CD4^+ ^cell percentage below 25%. Approximately half the HIV-positive children with oedema had CD4^+ ^counts below 25%. Very low CD4^+ ^cell percentages consistent with a laboratory diagnosis of AIDS have rarely been described in HIV-uninfected children with or without mixed infections. Reports on the proportions of T cell and CD4^+ ^cell percentages in severely malnourished children have shown inconsistent findings [3,4,19,20]. The difference in the results may be influenced by the differences in study designs and sample size.

Alterations in the haematological functions in malnutrition have been documented [17]. A recent study has reported cases of 5 malnourished children with mixed infection in whom the monocyte counts were higher than those in 4 malnourished children with only respiratory infection although their HIV status was not reported [3]. Therefore, both granulocyte and lymphocyte suppression observed in this study is an indication of reduced haemopoietic function, and the additional burden of HIV-1 infection appears to further reduce this function.

The CD4^+ ^cell percentages in this study were lower in children who presented with non-oedematous severe malnutrition, and this finding was consistent in both HIV-infected and uninfected groups. Earlier studies reported that oedematous malnutrition had lower T cell counts [19,21], while others found no difference in the T cell count with regard to the type of malnutrition [4]. The reason for these controversies is unclear. The only known fact is that severe malnutrition alters the immunological competence through a number of mechanisms, including apoptosis of the thymus gland [22, 23] and micronutrient deficiencies [24]. Likewise, the rapid destruction of the CD4^+ ^T lymphocytes by the HIV-1 virus has been well established. However, mechanisms leading to cellular immunological alterations in cases wherein severe malnutrition and HIV-1 virus infection co-exist are yet unclear.

It is interesting to notice that the HIV-positive children less often present with oedema, that is, oedema was present in just over 40% of the HIV-positive children, while it was seen in over 60% of the HIV-negative children. Severe wasting in the absence of oedema is a common feature observed in severe malnutrition with concurrent HIV infection [6,11,13-15]. The novel observation of this study was that the CD4^+ ^percentages were lower in both HIV-positive and HIV-negative children without oedema than in children with oedema. Both the above-mentioned observations appear to imply that the development of oedema requires a certain degree of immunocompetence, which is an interesting clue to the pathophysiology of oedema in severe malnutrition.

## Conclusion

Severe protein energy malnutrition is associated with the depletion of the haematological and lymphocyte subsets, and this depletion is exacerbated by the presence of HIV-1 infection. Cell-mediated immunosuppression is more marked in non-oedematous severe malnutrition, regardless of the HIV status.

## Competing interests

The author(s) declare that they have no competing interests.

## Authors' contributions

All authors have participated in the design of the study, interpretation of the results, statistical analysis and writing of the manuscript. HB supervised patient recruitment, follow-up and data collection. All authors have read and approved the final manuscript.

## References

[B1] Chandra RK (1999). Nutrition and immunology: from the clinic to cellular biology and back again. Proc Nutr Soc.

[B2] Scrimshaw NS (1997). Synergism of nutrition, infection, and immunity: an overview. Am J Clin Nutr.

[B3] Najera O (2004). Flow cytometry study of lymphocyte subsets in malnourished and well-nourished children with bacterial infections. Clin Diagn Lab Immunol.

[B4] Rikimaru T (1998). Humoral and cell-mediated immunity in malnourished children in Ghana. Eur J Clin Nutr.

[B5] Bakaki P (2001). Epidemiologic and clinical features of HIV-infected and HIV-uninfected Ugandan children younger than 18 months. J Acquir Immune Defic Syndr.

[B6] Ticklay IM (1997). HIV infection in malnourished children in Harare, Zimbabwe. East Afr Med J.

[B7] Ndugwa CM (1988). Uganda: paediatric AIDS. AIDS Action.

[B8] Bhuta UM, Ulstein H (2003). Evaluation of the Beckman Coulter AcT 5 diff AL hematology analyzer in a hospital setting. Lab Hematol.

[B9] WHO , World Health Organization (1999). Management of severe malnutrition: a manual for physicians and other senior health workers. Management of severe malnutrition.

[B10] Document WHO, World Health Organization (1993). Guidelines for the Clinical Management of HIV infection in children.. WHO/GPA/IDS/HCS/933.

[B11] Lugada ES (2004). Population-based hematologic and immunologic reference values for a healthy Ugandan population. Clin Diagn Lab Immunol.

[B12] Fakhir S (1989). Cell-mediated immune responses in malnourished host. J Trop Pediatr.

[B13] Carney JM (1980). Cell Mediated defects and infection, A study of malnourished hospitalized children. Am J Dis Child.

[B14] Borelli P (2004). Haematological alterations in protein malnutrition. Rev bras hematol hemoter.

[B15] Keusch GT (2003). The history of nutrition: malnutrition, infection and immunity. J Nutr.

[B16] Savino W (2002). The Thymus. European Journal of Clinical Nutrition.

[B17] Chevalier P (1996). Immuno-nutritional recovery of children of children with severe malnutrition. Sante.

[B18] Zaman K (1997). Malnutrition, cell-mediated immune deficiency and acute upper respiratory infections in rural Bangladeshi children. Acta Paediatr.

[B19] Prazuck T (1993). HIV infection and severe malnutrition: a clinical and epidemiological study in Burkina Faso. AIDS.

[B20] Macallan DC (1999). Nutrition and immune function in human immunodeficiency virus infection.

[B21] Kessler L (2000). The impact of the human immunodeficiency virus type 1 on the management of severe malnutrition in Malawi. Ann Trop Paediatr.

[B22] Embree J (2001). Lymphocyte subsets in human immunodeficiency virus type 1-infected and uninfected children in Nairobi. Pediatr Infect Dis J.

